# Glucose Enhances Leptin Signaling through Modulation of AMPK Activity

**DOI:** 10.1371/journal.pone.0031636

**Published:** 2012-02-16

**Authors:** Haoran Su, Lin Jiang, Christin Carter-Su, Liangyou Rui

**Affiliations:** Department of Molecular and Integrative Physiology, University of Michigan Medical School, Ann Arbor, Michigan, United States of America; University of Texas Health Science Center at San Antonio, United States of America

## Abstract

Leptin exerts its action by binding to and activating the long form of leptin receptors (LEPRb). LEPRb activates JAK2 that subsequently phosphorylates and activates STAT3. The JAK2/STAT3 pathway is required for leptin control of energy balance and body weight. Defects in leptin signaling lead to leptin resistance, a primary risk factor for obesity. Body weight is also regulated by nutrients, including glucose. Defects in glucose sensing also contribute to obesity. Here we report crosstalk between leptin and glucose. Glucose starvation blocked the ability of leptin to stimulate tyrosyl phosphorylation and activation of JAK2 and STAT3 in a variety of cell types. Glucose dose-dependently enhanced leptin signaling. In contrast, glucose did not enhance growth hormone-stimulated phosphorylation of JAK2 and STAT5. Glucose starvation or 2-deoxyglucose-induced inhibition of glycolysis activated AMPK and inhibited leptin signaling; pharmacological inhibition of AMPK restored the ability of leptin to stimulate STAT3 phosphorylation. Conversely, pharmacological activation of AMPK was sufficient to inhibit leptin signaling and to block the ability of glucose to enhance leptin signaling. These results suggest that glucose and/or its metabolites play a permissive role in leptin signaling, and that glucose enhances leptin sensitivity at least in part by attenuating the ability of AMPK to inhibit leptin signaling.

## Introduction

Leptin is a metabolic hormone that is required for the maintenance of normal energy balance and body weight [1]. Leptin is secreted into the bloodstream from adipose tissues [2]. It suppresses food intake, increases energy expenditure, and promotes weight loss primarily by binding to and activating LEPRb in the hypothalamus [1]. Genetic deficiency of leptin or LEPRb results in hyperphagia and severe obesity in both rodents and humans [2], [3], [4], [5], [6]. However, circulating leptin levels increase under most obesity conditions [7], [8]; furthermore, diet-induced obesity is associated with a reduced ability of leptin to suppress both food intake and body weight gain [1], [7], [9]. Leptin resistance is believed to be the primary risk factor for obesity [1]; however, the underlying molecular mechanisms for leptin resistance remain largely unknown.

Defects in leptin signaling are likely to be a major contributor to leptin resistance [1]. LEPRb is a cytokine receptor family member that binds to Janus kinase 2 (JAK2), a cytoplasmic tyrosine kinase [1]. Upon leptin binding, LEPRb activates its associated JAK2 that in turn phosphorylates LEPRb at multiple tyrosines, including Tyr^985^, Tyr^1077^, and Tyr^1138^ [10], [11]. Phosphorylated Tyr^985^ binds to SHP2, leading to activation of the ERK pathway [11], [12], [13]. Brain-specific deletion of SHP2 results in leptin resistance and obesity, suggesting that the LEPRb/SHP2/ERK pathway is required for a full leptin action [14]. Phospho-Tyr^985^ also binds to SOCS3, which negatively regulates leptin signaling [15]. Phospho-Tyr^1077^ binds to Signal Transducer and Activator of Transcription (STAT) 5 [16], [17]. Phospho-Tyr^1138^ recruits STAT3, thus allowing JAK2 to phosphorylate and activate STAT3 [12]. The JAK2/STAT3 pathway is the best-understood pathway in leptin action. Brain specific deletion of STAT3 results in leptin resistance and obesity in mice [18]. Replacement of Tyr^1138^ with Phe or Ala also results in severe leptin resistance and obesity [19], [20]. These observations indicate that the JAK2/STAT3 pathway is required for leptin regulation of energy balance and body weight.

Energy balance and body weight is also regulated by nutrients, including glucose [21]. Hypothalamic glucopenia (glucose deficit) promotes feeding [22], [23]. Glucose is a metabolic fuel that is metabolized to generate ATP through glycolysis and the tricarboxylic acid cycle. Glucose or its metabolites serve as biosynthetic precursors for lipids and various glycoproteins. Additionally, glucose and its metabolites are also involved in cell signaling. Glucose inhibits K_ATP_ channels in hypothalamic neurons, thus increasing cell excitability [24], [25]. Glucose starvation stimulates 5′-AMP-activated protein kinase (AMPK), a molecular energy sensor, in many cell types, including hypothalamic neurons [26], [27]. Glucose-sensing neurons are located in multiple regions in the central nervous system (CNS), including the hypothalamus, brain stem, and amygdala, and hypothalamic glucose sensing is believed to play an important role in energy homeostasis [21], [28]. Glucose directly excites POMC neurons, but inhibits AgRP neurons, in the arcuate nucleus of the hypothalamus [28]. Leptin also stimulates POMC neurons and inhibits AgRP neurons, two subpopulations of hypothalamic neurons that play a key role in the control of energy balance and body weight [1]. Glucose regulates not only the excitability of glucose-sensing neurons but also biochemical events in these cells. In POMC neurons, glucose increases the levels of hypoxia-inducible factors that in turn stimulate POMC expression and reduce body weight [29]. Therefore, hypothalamic neurons are likely to integrate signals from both leptin and glucose, thus controlling energy balance and body weight. In the current study, we show that glucose plays a permissive role in leptin signaling. Glucose promotes leptin pathways at least in part by inhibiting AMPK.

## Results

### Glucose plays a permissive role in leptin stimulation of STAT3 phosphorylation

**Figure 1:**
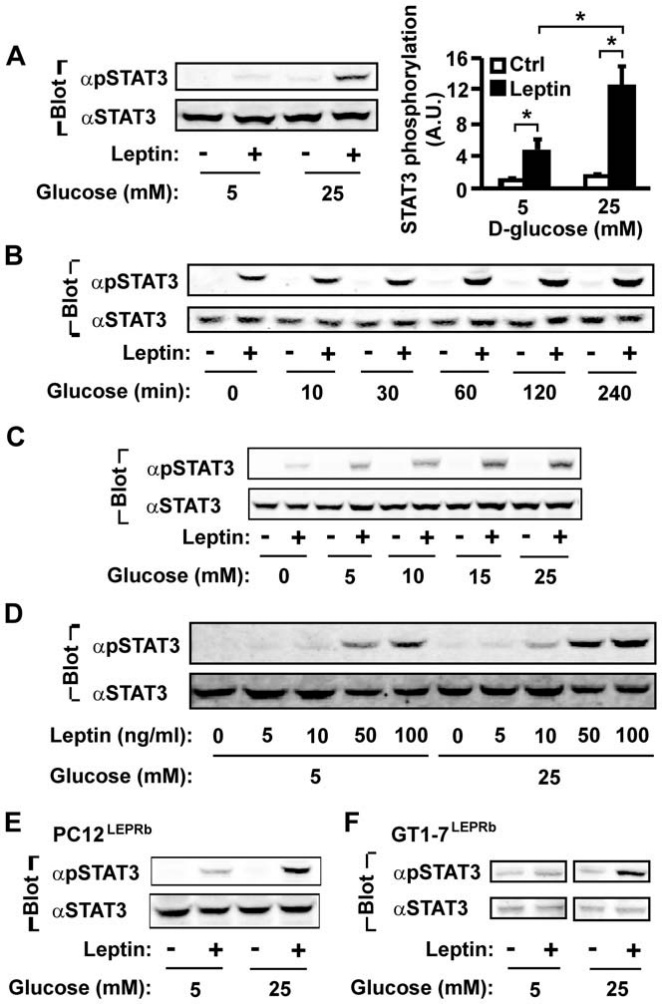
Glucose enhances leptin stimulation of STAT3 phosphorylation. *A*, γ2A^LEPRb/JAK2^ cells were grown overnight in serum-free medium supplemented with 5 or 25 mM glucose and treated with 100 ng/ml leptin for 10 min. Cell extracts were immunoblotted with anti-phospho-STAT3 (pTyr^705^) (αpSTAT3) or αSTAT3 antibodies. The amounts of phospho-STAT3 and total STAT3 were quantified using densitometry, and STAT3 phosphorylation was normalized to the total amount of STAT3. *P<0.05. *B*, γ2A^LEPRb/JAK2^ cells were deprived of serum in the presence of 5 mM glucose overnight. Cells were pretreated with 25 mM glucose for 0, 10, 30, 60, 120 or 240 min, and then treated with 100 ng/ml leptin for 10 min. Cell extracts were immunoblotted with αpSTAT3 or αSTAT3. *C*, γ2A^LEPRb/JAK2^ cells were deprived of serum overnight in the presence of 0, 5, 10, 15 or 25 mM glucose, and stimulated with 100 ng/ml leptin for 10 min. Cell extracts were immunoblotted with αpSTAT3 or αSTAT3. *D*, γ2A^LEPRb/JAK2^ cells were deprived of serum in the presence of 5 or 25 mM glucose overnight, and then stimulated with leptin for 10 min at various concentrations. Cell extracts were immunoblotted with αpSTAT3 or αSTAT3, respectively. *E–F*, PC12^LEPRb^ neurons (*E*) and GT1-7^LEPRb^ cells (*F*) were deprived of serum overnight in 5 or 25 mM glucose and then treated with 100 ng/ml leptin for 10 min. Cell extracts were immunoblotted with αpSTAT3 or αSTAT3.

We have generated and characterized human fibrosarcoma (γ2A^LEPRb/JAK2^) that stably expresses LEPRb and JAK2 [30]. To determine whether glucose levels affect leptin signaling, γ2A^LEPRb/JAK2^ cells were grown overnight in serum-free medium supplemented with either 5 mM or 25 mM D-glucose and treated with 100 ng/ml leptin for 10 min. Tyrosyl phosphorylation of STAT3 in cell extracts was examined by immunoblotting with anti-phospho-STAT3 (pTyr^705^) antibody (αpSTAT3). At 5 mM glucose, leptin modestly stimulated STAT3 phosphorylation; in contrast, leptin robustly stimulated STAT3 phosphorylation at 25 mM glucose (Fig. 1A). Glucose enhanced leptin signaling in both time- and dose-dependent manners. Glucose enhancement was detected within 30 min and progressively increased for 4 h (Fig. 1B). Leptin-stimulated STAT3 Tyr^705^ phosphorylation also increased progressively from 5–25 mM glucose (Fig. 1C). To determine whether glucose increases leptin sensitivity, we examined leptin dose-responses at 5 or 25 mM D-glucose. Glucose increased leptin-stimulated STAT3 Tyr^705^ phosphorylation at both low (5–10 ng/ml) and high concentrations (50–100 ng/ml) of leptin (Fig. 1D). These data suggest that glucose increases both leptin sensitivity and maximal responses.

To determine whether glucose similarly enhances leptin signaling in neurons, PC12^LEPRb^ cells, which stably express LEPRb [31], were differentiated into neurons with nerve growth factor (NGF). The neurons were grown overnight in serum-free medium supplemented with 5 or 25 mM D-glucose and stimulated with 100 ng/ml leptin for 10 min. Leptin stimulated STAT3 Tyr^705^ phosphorylation modestly at 5 mM glucose but robustly at 25 mM glucose (Fig. 1E). Further reduction in glucose (3 mM) largely abolished leptin signaling (data not shown). To determine whether glucose enhances leptin signaling in hypothalamic neurons, LEPRb was stably introduced into GT1-7 cells (GT1-7^LEPRb^), a mouse hypothalamic line [32], and leptin signaling was examined at low or high levels of D-glucose. At 5 mM glucose, leptin barely stimulated STAT3 Tyr^705^ phosphorylation; in contrast, leptin robustly stimulated STAT3 phosphorylation at 25 mM glucose (Fig. 1F). Together, these results demonstrate that glucose plays a permissive role in leptin signaling and dose-dependently enhances leptin signaling.

### Glucose enhances leptin stimulation of JAK2

**Figure 2:**
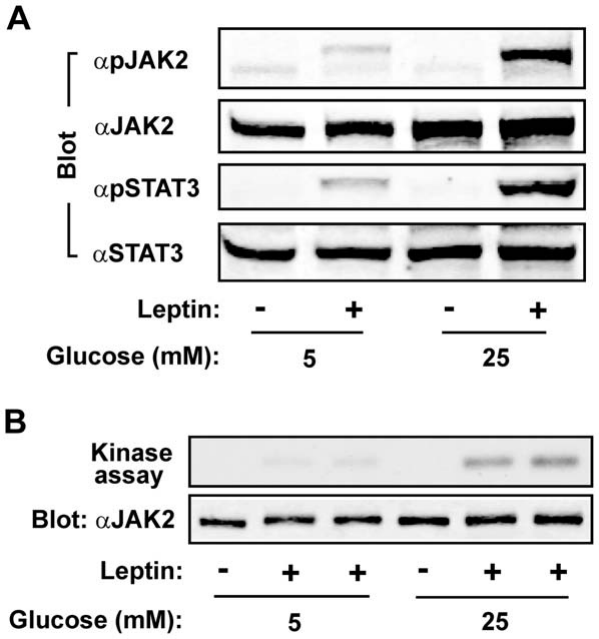
Glucose enhances leptin stimulation of JAK2. *A*, γ2A^LEPRb/JAK2^ cells were deprived of serum in the presence of 5 or 25 mM glucose overnight and then treated with 100 ng/ml leptin for 10 min. Cell extracts were immunoblotted with anti-phospho-JAK2 (pTyr^1007/1008^) (αpJAK2), αJAK2, αpSTAT3, or αSTAT3 as designated. *B*, γ2A^LEPRb/JAK2^ cells were treated with 5 or 25 mM glucose overnight and then with 100 ng/ml leptin for 10 min. JAK2 in cell extracts was immunoprecipitated with αJAK2 and subjected to an *in vitro* kinase assay. The same blots were immunoblotted with αJAK2.

Leptin stimulates STAT3 phosphorylation by both JAK2-dependent and -independent mechanisms [31]. To determine whether glucose is involved in leptin stimulation of JAK2, γ2A^LEPRb/JAK2^ cells were incubated overnight with either 5 mM or 25 mM D-glucose and treated with 100 ng/ml leptin for 10 min. Cell extracts were immunoblotted with anti-phospho-JAK2 (pTyr^1007/1008^) antibody. Phosphorylation of Tyr^1007^ in the activation loop is required for JAK2 activation [33], [34]. 25 mM glucose markedly increased the ability of leptin to stimulate tyrosyl phosphorylation of both JAK2 and STAT3, compared with 5 mM glucose (Fig. 2A). Additionally, JAK2 was immunopurified and its catalytic activity was measured using an *in vitro* kinase assay. Leptin barely activated JAK2 at 5 mM glucose but robustly stimulated JAK2 at 25 nM glucose (Fig. 2B). These results further support the conclusion that glucose plays a permissive role in leptin signaling.

### Glucose differentially enhances leptin- but not growth hormone (GH)-stimulated JAK2/STAT pathway

**Figure 3:**
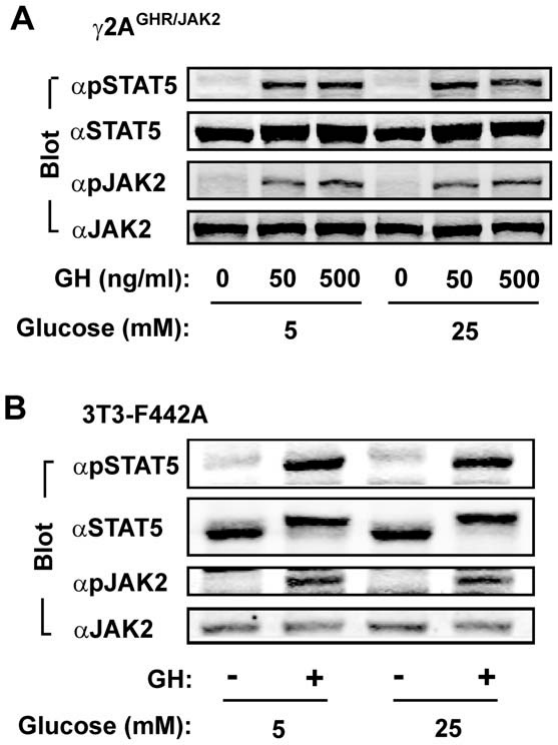
Glucose does not enhance GH-stimulated phosphorylation of JAK2 and STAT5. *A*, γ2A^GHR/JAK2^ cells were deprived of serum in the presence of 5 or 25 mM glucose overnight and then treated with GH for 15 min. Cell extracts were immunoblotted with αpSTAT5, αSTAT5, αpJAK2 or αJAK2, respectively. *B*, 3T3-F442A cells were treated with 5 or 25 mM glucose overnight and then with 50 ng/ml GH for 15 min. Cell extracts were immunoblotted with the indicated antibodies.

To determine whether glucose similarly enhances the JAK/STAT pathway in response to other hormones, we generated γ2A^GHR/JAK2^ cells that stably express GH receptors (GHR) and JAK2 [31]. JAK2 binds to GHR and mediates STAT5 tyrosyl phosphorylation and activation in response to GH [35], [36]. γ2A^GHR/JAK2^ cells were pretreated with 5 mM or 25 mM D-glucose and stimulated with GH for 15 min. Cell extracts were immunoblotted with anti-phospho-STAT5 (pTyr694 of STAT5a or pTyr699 of STAT5b) or anti-phospho-JAK2 (pTyr^1007/1008^). GH stimulated phosphorylation of both STAT5 and JAK2; surprisingly, glucose did not increase the ability of GH to stimulate phosphorylation of either STAT5 or JAK2 (Fig. 3A). To determine whether glucose enhances endogenous GHR/JAK2/STAT5 pathway, 3T3-F442A cells were grown in the presence of either 5 mM or 25 mM D-glucose and treated with GH. GH stimulated phosphorylation of STAT5 and JAK2 to a similar degree between 5 mM and 25 mM glucose (Fig. 3B). Thus, glucose starvation specifically suppresses leptin stimulation of the JAK2/STAT3 pathway but not GH stimulation of the JAK2/STAT5 pathway. These data also suggest that glucose starvation-induced suppression of leptin signaling is unlikely to be caused by nonspecific inhibition of cell signaling and JAK/STAT pathways.

### Glycolysis is required for glucose enhancement of leptin signaling

**Figure 4:**
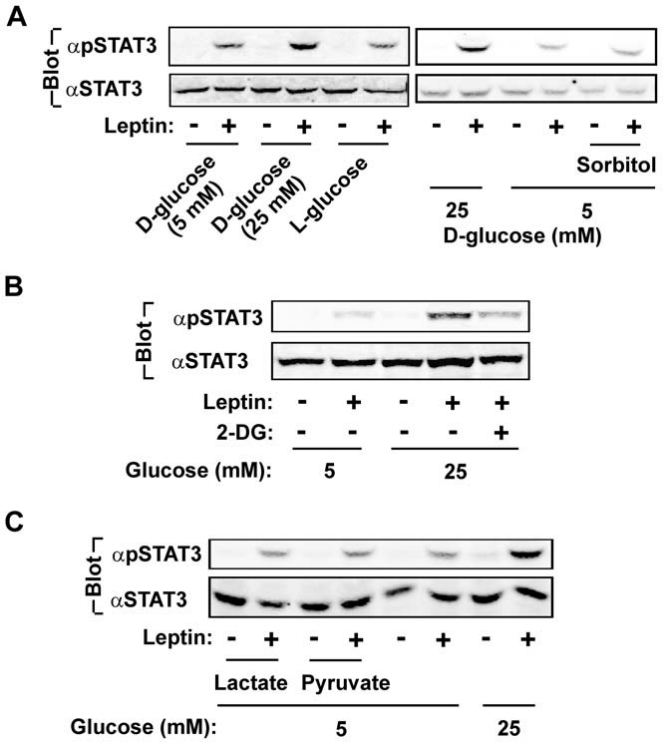
Glycolysis is required for glucose to enhance leptin signaling. *A*, γ2A^LEPRb/JAK2^ cells were grown overnight in serum-free medium supplemented with 25 mM D-glucose, 5 mM D-glucose plus 20 mM L-glucose, or 5 mM D-glucose plus 20 mM sorbitol. Cells were stimulated with 100 ng/ml leptin for 10 min, and cell extracts were immunoblotted with αpSTAT3 or αSTAT3. *B*, γ2A^LEPRb/JAK2^ cells were grown overnight in serum-free medium supplemented with 25 mM D-glucose, pretreated with 25 mM 2-DG for 3 h, and then treated with 100 ng/ml leptin for 10 min. Cell extracts were immunoblotted with αpSTAT3 or αSTAT3. *C*. γ2A^LEPRb/JAK2^ cells were grown overnight (∼15 h) in serum-free medium containing 5 mM D-glucose plus additional 20 mM lactate, pyruvate, or D-glucose, and stimulated with 100 ng/ml leptin for 10 min. Cell extracts were immunoblotted with αpSTAT3 or αSTAT3.

To exclude osmolarity as a contributing factor, γ2A^LEPRb/JAK2^ cells were grown in 5 mM D-glucose supplemented with additional 20 mM L-glucose, sorbitol, or D-glucose, and stimulated with leptin. D-glucose, but not L-glucose or sorbitol, increased the ability of leptin to stimulate STAT3 Tyr^705^ phosphorylation (Fig. 4A). L-glucose is unable to be metabolized, raising the possibility that glycolysis is important for glucose to enhance leptin signaling. To test this idea, cells were pretreated with 25 mM D-glucose in the presence or absence of 2-deoxyglucose (2-DG), which blocks glycolysis. 2-DG prevented D-glucose from enhancing leptin stimulation of STAT3 phosphorylation (Fig. 4B). In contrast to D-glucose, neither lactate nor pyruvate enhanced leptin signaling (Fig. 4C). Both lactate and pyruvate are oxidized via the tricarboxylic acid cycle; thus, the tricarboxylic acid cycle is not sufficient to enhance leptin signaling. Therefore, glycolysis is likely to play a permissive role for leptin signaling.

### AMPK mediates glucose regulation of leptin signaling

**Figure 5:**
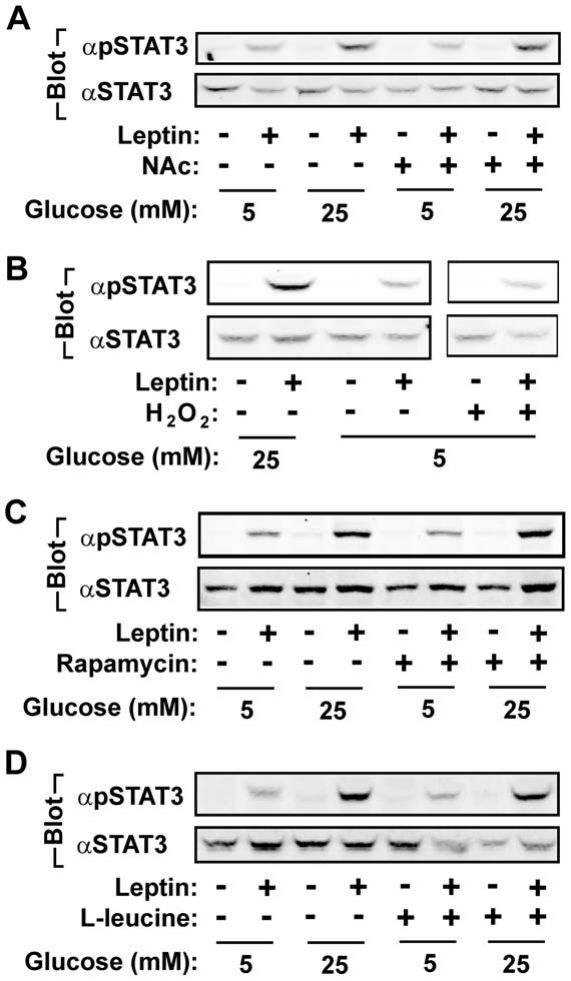
Oxidative stress, the mTOR pathway, and the p38 MAPK pathway do not mediate glucose enhancement of leptin signaling. *A–B*. γ2A^LEPRb/JAK2^ cells were grown overnight in serum-free medium supplemented with 5 or 25 mM D-glucose, 10 mM NAc (A) or 200 µM H_2_O_2_ (B). Cells were stimulated with 100 ng/ml leptin for 10 min, and cell extracts were immunoblotted with αpSTAT3 or αSTAT3. *C–D*. γ2A^LEPRb/JAK2^ cells were incubated in 5 or 25 mM glucose overnight, pretreated with 50 nM rapamycin for 1 h (C) or 2 mM L-leucine for 2 h (D), and then stimulated with 100 ng/ml leptin for 10 min. Cell extracts were immunoblotted with αpSTAT3 or αSTAT3.

Glucose metabolism links to oxidative stress and the activation of the mTOR pathway. To determine whether reactive oxygen species (ROS) mediate glucose action on leptin signaling, γ2A^LEPRb/JAK2^ cells were treated with 25 mM D-glucose in the presence of the antioxidant N-acetyl cysteine (NAc) (10 mM). NAc did not inhibit the ability of glucose to enhance leptin-stimulated Tyr^705^ STAT3 phosphorylation (Fig. 5A). Additionally, H_2_O_2_ was unable to mimic glucose action to enhance leptin signaling (Fig. 5B). To examine the mTOR pathway, cells were pretreated with rapamycin, a TORC1 inhibitor. Rapamycin did not decrease the ability of glucose to enhance leptin-stimulated STAT3 phosphorylation (Fig. 5C). In agreement, L-leucine, a potent activator of mTOR, was also unable to enhance leptin signaling (Fig. 5D). These data suggest that oxidative stress and the TORC1 pathway are unlikely to mediate glucose enhancement of leptin signaling.

**Figure 6:**
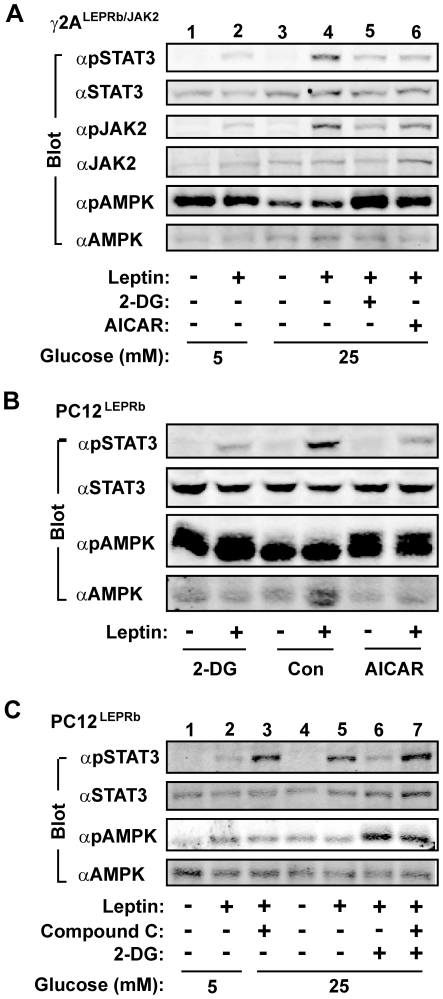
AMPK is involved in glucose enhancement of leptin signaling. *A*, γ2A^LEPRb/JAK2^ cells were deprived of serum overnight (in 25 mM glucose). Cells were treated with 25 mM 2-DG or 2 mM AICAR for 3 h, and then with 100 ng/ml leptin for additional 10 min. Cell extracts were immunoblotted with the indicated antibodies. *B*, PC12^LEPRb^ neurons were deprived of serum overnight in the presence of 25 mM glucose, and treated with 25 mM 2-DG or 2 mM AICAR for 1 h and then with 100 ng/ml leptin for additional 10 min. Cell extracts were immunoblotted with the indicated antibodies. *C*, PC12^LEPRb^ neurons were incubated overnight (∼15 h) in the presence or absence of 40 µM compound C. Some cells were pretreated with 2-DG (25 mM) for 1 h as indicated. Cells were stimulated with 100 ng/ml leptin for 10 min, and cell extracts were immunoblotted with the indicated antibodies.

Inhibition of glycolysis by either glucose starvation or 2-DG treatments activates AMPK [37], [38], raising the possibility that AMPK may be involved in D-glucose deficiency-induced suppression of leptin signaling. To test this idea, γ2A^LEPRb/JAK2^ cells were treated with AICAR, an AMPK activator. As shown in Figures 1A and 2A, 25 mM D-glucose increased leptin-stimulated tyrosyl phosphorylation of STAT3 and JAK2 compared with 5 mM glucose (Fig. 6A, lane 4). Importantly, both 2-DG and AICAR strongly attenuated the ability of glucose to enhance leptin signaling (Fig. 6A, lanes 5–6). Both 2-DG and AICAR stimulated AMPK phosphorylation (pThr^172^) (Fig. 6A, panel 5); D-glucose deficiency also led to AMPK phosphorylation (Fig. 6A, lanes 1–2, panel 5). AMPK phosphorylation on Thr^172^ is required for AMPK activity and serves as a marker for estimating its activation [26]. These data suggest that AMPK activation inhibits leptin stimulation of the JAK2/STAT3 pathway, and that glucose provides a permissive condition for leptin signaling at least in part by inhibiting AMPK.

To determine whether AMPK inhibits leptin signaling in neurons, PC12^LEPRb^ cells were differentiated into neurons. Both AICAR and 2-DG suppressed leptin-stimulated tyrosyl phosphorylation of STAT3 at 25 mM glucose in PC12^LEPRb^ neurons (Fig. 6B). To determine whether inhibition of AMPK improves leptin signaling under low glucose conditions, PC12^LEPRb^ neurons were pretreated with compound C, a commonly used AMPK inhibitor, in the presence of 5 mM glucose. Compound C markedly increased the ability of leptin to stimulate STAT3 phosphorylation (Fig. 6C, lanes 2–3). At 25 mM D-glucose, 2-DG suppressed leptin-stimulated phosphorylation of STAT3 (Fig. 6C, lanes 5–6), and compound C reversed 2-DG suppression of leptin signaling (Fig. 6C, lane 7). These data further confirm that AMPK inhibits leptin stimulation of the JAK2/STAT3 pathway, and that glucose enhances leptin signaling at least in part by inhibiting AMPK.

## Discussion

Leptin resistance is the primary risk factor for obesity [1]. Upregulation of a number of negative regulators of leptin signaling, including PTP1b and SOCS3, is likely to contribute to leptin resistance [39], [40], [41], [42], [43]. Conversely, downregulation of positive regulators, including SH2B1, may also contribute to leptin resistance [44], [45]. Thus, it is important to identify additional regulators of leptin signaling in order to fully understand energy homeostasis and body weight regulation. Here we have identified glucose and AMPK as new regulators of leptin sensitivity.

Both leptin and glucose, acting on hypothalamic neurons, suppress food intake and weight gain; conversely, leptin deficiency and brain glucopenia promote hyperphagia and weight gain [1], [21]. However, it is unclear whether leptin and glucose exhibit crosstalk in hypothalamic neurons. In this study, we report that glucose is likely to play a permissive role in leptin signaling and to improve leptin sensitivity in a dose-dependent manner. We showed that D-glucose deficiency blocks the ability of leptin to stimulate tyrosyl phosphorylation of JAK2 and STAT3. Additionally, D-glucose enhances leptin signaling in a variety of cell types, including human γ2A fibrosarcoma cells, rat PC12 neurons, and murine GT1-7 hypothalamic neurons. Glycolysis appears to be required for leptin signaling, since blocking glycolysis suppressed leptin signaling and decreased the ability of glucose to increase leptin stimulation of JAK2 and STAT3 phosphorylation. Surprisingly, glucose did not increase the ability of GH to stimulate the JAK2/STAT5 pathway. These observations raise the possibility that glucose or its metabolites may facilitate the coupling between LEPRb and JAK2 but not between GHR and JAK2.

We also identified AMPK as a potential negative regulator of leptin sensitivity. AMPK is a molecular energy sensor that is activated by AMP (associated with low intracellular energy levels) and a variety of hormones and cytokines [26], [27]. Resembling leptin resistance, activation of hypothalamic AMPK increases food intake and body weight in mice; in contrast, blocking hypothalamic AMPK increases the anorexigenic effect of leptin [46], [47]. We observed that at high levels of glucose (25 mM), AMPK activity was suppressed and pharmacological activation of AMPK by either 2-DG or ARCAR markedly reduced the ability of leptin to stimulate tyrosyl phosphorylation of JAK2 and STAT3. Conversely, at low levels of glucose, AMPK was highly activated and leptin signaling was suppressed; pharmacological inhibition of AMPK robustly enhanced leptin signaling. These results suggest that glucose plays a permissive role in the maintenance of normal leptin sensitivity by suppressing AMPK and reversing AMPK inhibition of leptin signaling. Additionally, these observations raise the possibility that AMPK may also mediate crosstalk between leptin and other factors, in addition to glucose, that regulate the AMPK pathway.

In summary, we described novel crosstalk between leptin and glucose. Glycolysis provided a permissive condition for leptin to stimulate the JAK2/STAT3 pathway. We have also identified AMPK as a potential negative regulator of leptin signaling. Glucose enhanced leptin signaling at least in part by inhibiting the ability of AMPK to suppress leptin signaling.

## Materials and Methods

### Materials

Antibodies against phospho-STAT3(pTyr^705^), STAT3, STAT5, and AMPKα were purchased from Santa Cruz Biotechnology Inc. (Santa Cruz, CA). Anti-phospho-AMPK (pThr^172^) antibody was from Cell Signaling Technology Inc. (Beverly, MA). Anti-JAK2 antibody was from Millipore Corp. (Bedford, MA). Anti-phospho-JAK2 (pTyr^1007/1008^) and anti-phospho-tyrosine (pY20) antibodies were from Upstate Biotechnology Inc. (Lake Placid, NY). Anti-phospho-STAT5 antibody was obtained from Zymed Labs, Inc. (San Francisco, CA). 5-aminoimidazole-4-carboxamide 1-β-D-ribofuranoside (AICAR) and SB203580 were from Calbiochem Bioscience Inc. (La Jolla, CA). Recombinant mouse leptin was from the National Hormone and Peptide Program, NIDDK, National Institutes of Health (Torrance, CA). Compound C and nerve growth factor were from Sigma-Aldrich.

### Cell Culture, Neuronal Differentiation, and Immunoblotting

γ2A^LEPRb/JAK2^, γ2A^GHR/JAK2^ and PC12^LEPRb^ cell lines have been described previously [31]. GT1-7^LEPRb^ cell lines were derived from mouse hypothalamic tumor cells (GT1-7) [32]. LEPRb was stably introduced into GT1-7 cells via LEPRb retroviral infection [31]. γ2A^LEPRb/JAK2^ and γ2A^GHR/JAK2^ cells were cultured in Dulbecco's modified Eagle's medium (DMEM) containing 5% heat-inactivated fetal bovine serum (FBS) in the presence of 25 mM glucose, 100 units/ml penicillin and 100 µg/ml streptomycin at 37°C in a humidified atmosphere of 5% CO_2_. PC12^LEPRb^ cells were grown on collagen-coated dishes at 37°C in 5% CO_2_ in DMEM supplemented with 25 mM glucose, 100 units/ml penicillin, 100 µg/ml streptomycin, 10% heat-inactivated horse serum, and 5% FBS. To induce neuronal differentiation, PC12^LEPRb^ cells were cultured for 3 days in DMEM supplemented with 25 mM glucose, 2% horse serum, 1% FBS, 100 ng/ml NGF, 100 units/ml penicillin, and 100 µg/ml streptomycin. For immunoblotting, cells were deprived of serum overnight in DMEM containing 0.6% bovine serum albumin (BSA) in the presence of 5 mM D-glucose supplemented with additional 20 mM D-glucose, L-glucose, sorbitol, pyruvate, or lactate. Cells were pretreated with or without the indicated compounds, and then treated with leptin. Cell extracts were prepared as described previously [48], and immunoblotted with the indicated antibodies. Blots were visualized using Odyssey Infrared Imaging System (LI-COR Biosciences, Lincoln, NE).

### In Vitro Kinase Assays

JAK2 in cell extracts was immunoprecipitated with anti-JAK2 antibody and absorbed on protein A-agarose beads. The beads were washed extensively with a kinase reaction buffer (50 mM HEPES, pH 7.6, 10 mM MnCl_2_, 100 mM NaCl, 0.5 mM dithiothreitol, 1 mM Na_3_VO_4_) and then incubated at room temperature for 30 min in the kinase reaction buffer supplemented with [γ-^32^P]ATP (10 µCi), 20 µM cold ATP, 10 µg/ml aprotinin, and 10 µg/ml leupeptin. The kinase reaction was stopped by washing the beads with lysis buffer. JAK2 was eluted from the beads by boiling for 5 min in the SDS-PAGE sample buffer. JAK2 was resolved by SDS-PAGE and transferred onto nitrocellulose membranes. JAK2 autophosphorylation was detected by autoradiography. The same blots were immunoblotted with anti-JAK2 antibody.

### Statistical Analysis

Data are presented as means ± SEM. Differences between groups were analyzed by two-tailed Student's t test. *P*<0.05 was considered statistically significant.
